# Pan-azole- and multi-fungicide-resistant *Aspergillus fumigatus* is widespread in the United States

**DOI:** 10.1128/aem.01782-23

**Published:** 2024-04-01

**Authors:** B. N. Celia-Sanchez, B. Mangum, L. F. Gómez Londoño, C. Wang, B. Shuman, M. T. Brewer, M. Momany

**Affiliations:** 1 Department of Plant Biology, University of Georgia, Athens, Georgia, USA; 2 Department of Plant Pathology, University of Georgia, Athens, Georgia, USA; Centers for Disease Control and Prevention, Atlanta, Georgia, USA

**Keywords:** antifungal resistance

## Abstract

*Aspergillus fumigatus* is an important global fungal pathogen of humans. Azole drugs are among the most effective treatments for *A. fumigatus* infection. Azoles are also widely used in agriculture as fungicides against fungal pathogens of crops. Azole-resistant *A. fumigatus* has been increasing in Europe and Asia for two decades where clinical resistance is thought to be driven by agricultural use of azole fungicides. The most prevalent mechanisms of azole resistance in *A. fumigatus* are tandem repeats (TR) in the *cyp51A* promoter coupled with mutations in the coding region which result in resistance to multiple azole drugs (pan-azole resistance). Azole-resistant *A. fumigatus* has been isolated from patients in the United States (U.S.), but little is known about its environmental distribution. To better understand the distribution of azole-resistant *A. fumigatus* in the U.S., we collected isolates from agricultural sites in eight states and tested 202 isolates for sensitivity to azoles. We found azole-resistant *A. fumigatus* in agricultural environments in seven states showing that it is widespread in the U.S. We sequenced environmental isolates representing the range of U.S. sample sites and compared them with publicly available environmental worldwide isolates in phylogenetic, principal component, and ADMIXTURE analyses. We found worldwide isolates fell into three clades, and TR-based pan-azole resistance was largely in a single clade that was strongly associated with resistance to multiple agricultural fungicides. We also found high levels of gene flow indicating recombination between clades highlighting the potential for azole-resistance to continue spreading in the U.S.

**IMPORTANCE:**

*Aspergillus fumigatus* is a fungal pathogen of humans that causes over 250,000 invasive infections each year. It is found in soils, plant debris, and compost. Azoles are the first line of defense antifungal drugs against *A. fumigatus*. Azoles are also used as agricultural fungicides to combat other fungi that attack plants. Azole-resistant *A. fumigatus* has been a problem in Europe and Asia for 20 years and has recently been reported in patients in the United States (U.S.). Until this study, we did not know much about azole-resistant *A. fumigatus* in agricultural settings in the U.S. In this study, we isolated azole-resistant *A. fumigatus* from multiple states and compared it to isolates from around the world. We show that *A. fumigatus* which is resistant to azoles and to other strictly agricultural fungicides is widespread in the U.S.

## INTRODUCTION

*Aspergillus fumigatus* is an opportunistic fungal pathogen of humans found in soils, plant debris, and compost around the world. Causing over 250,000 invasive aspergillosis cases globally each year in immunocompromised individuals, *A. fumigatus* has a mortality rate approaching 90% with antimicrobial resistance (1, 2). In recognition of its clinical importance, the World Health Organization has designated *A. fumigatus* one of four critical fungal pathogens (1).

Infections caused by *A. fumigatus* are often treated with azole drugs (3). Azoles are also widely used as agricultural fungicides to combat fungal pathogens of crops (4). Azoles target a conserved Cyp51 protein (also known as Cyp51A, Cyp51B, and ERG11) in the pathway that makes ergosterol, a fungal sterol responsible for cell membrane fluidity (5). Binding of azoles to Cyp51 causes arrested growth, toxic sterol intermediates, and fungal cell death (6). *A. fumigatus* has evolved multiple resistance mechanisms to azoles including tandem repeats (TR) in the *cyp51A* promoter to increase expression coupled with point mutations that affect the binding of azoles. Tandem repeats of 34, 46, and 53 bases in the promoter coupled with point mutations in the coding region of *cyp51A* cause pan-azole resistance, defined as high levels of resistance to multiple azole drugs (7–9). The TR_34_/L98H and TR_46_/Y121F/T289A alleles are most associated with pan-azole resistance (10). Some isolates with TR in the *cyp51A* promoter have also been found to carry alleles for resistance to agricultural fungicides of the quinone outside inhibitor (QoI), benzimidazole (MBC), and succinate dehydrogenase inhibitor (Sdh) classes (11).

Previous studies have shown that worldwide *A. fumigatus* isolates are not structured geographically or by clinical or environmental source of isolation. However, two clades are often identified: Clade A, where isolates with multi-fungicide resistance and TR mutations causing azole resistance are clustered; and Clade B, which contains all other isolates (11–14). Recently, Lofgren et al. identified a third clade of *A. fumigatus* in a pan-genome analysis and renamed the existing clades with numbers: Clade 1 (Clade B), Clade 2 (Clade A), and Clade 3, which was previously called *A. fumigatus sensu stricto* (15, 16). Clade 3 contains mostly azole-sensitive isolates (16, 17).

Azole-resistant *A. fumigatus* has been recognized as a clinical problem in Europe and Asia for two decades (18, 19). Abundant evidence strongly supports the view that agricultural use of azoles led to pan-azole clinical resistance (11, 14, 18), including genetic similarities in azole-resistant *A. fumigatus* isolates from agricultural settings where azoles are used as fungicides and from clinical settings where azoles are used as drugs (14) and the presence in clinical isolates of markers for resistance to fungicides only used in agriculture (11). Resistance to clinical azole drugs has been reported in the U.S (13), where azoles are also used as agricultural fungicides (4, 10); however, information about the presence of azole-resistant *A. fumigatus* in agricultural environments in the United States (U.S.) has been limited. Environmental sampling of a cull pile from a single agricultural site in Georgia yielded the first environmental TR *A. fumigatus* strains identified in the U.S. (20). This was followed by more widespread sampling in Florida and Georgia where 12 of the 123 isolates were found to be pan-azole resistant (11). Analysis of 179 U.S. isolates by the Centers for Disease Control and Prevention (CDC) including passive surveillance data from clinical settings in 38 states, 13 environmental isolates from a Georgia peanut pile and 2 historical isolates from the CDC collection showed that 26% of isolates were resistant to azoles (13). To better understand azole-resistant *A. fumigatus* in the U.S., we collected samples from agricultural settings in previously unsampled states on the east and west coasts, isolated *A. fumigatus*, tested isolates for sensitivity to azoles and for markers of agricultural fungicide resistance, and analyzed their genetic relationships with each other and with worldwide environmental and clinical isolates using whole-genome sequence data. We found azole-resistant *A. fumigatus* in field soil and plant debris from different locations and different crops cultivated on both the east and west coasts of the U.S. Our population genomic analyses support the existence of three clades with a strong association of TR-based azole resistance and multi-fungicide resistance with Clade 2. Moreover, we show recombination between all clades, which suggests that pan-azole resistance will keep spreading within U.S. populations of *A. fumigatus* as it has worldwide.

## MATERIALS AND METHODS

### Sampling

Sampling was conducted as previously described (11). Briefly, between November 2018 and October 2019, soil, plant debris, or compost was collected from 29 agricultural sites in the eastern U.S. (New York, Pennsylvania, Virginia, South Carolina, and Georgia) and from 23 agricultural sites in the western U.S. (Washington, Oregon, and California; Table 1). Soil and compost were sampled by taking 3–5 soil cores to a depth of 10–15 cm. Fallen plant debris was collected with the soil if present. Collections were limited to four samples per site to minimize the isolation of clones. Samples were shipped overnight in sealed plastic bags and then stored at 4°C and with bags open to allow for gas exchange. Samples were generally processed within 2 weeks of collection.

### Isolation and storage

The samples were processed as described previously with some modifications (11, 18, 20). Briefly, 2 g of soil was suspended in 8 mL of 0.1 M sterile sodium pyrophosphate. Samples were vortexed for 30 seconds and allowed to settle for 1 minute. From the supernatant, 100 µL was pipetted onto 10 plates each of Sabouraud dextrose agar (SDA) control plates or SDA supplemented with 3 µg/mL of the fungicide tebuconazole or with 4 µg/mL of the antifungal itraconazole. All plates additionally contained 50 µg/mL of chloramphenicol and 5 µg/mL of gentamicin to inhibit bacterial growth and 25 µg/mL of Rose-Bengal dye to reduce the growth of *Rhizopus* and *Mucor*. The plates were sealed with micropore tape and incubated at 45°C for 48–72 hours. Colonies of *A. fumigatus* were identified by morphology and the distinctive gray-green spore color. Colonies that grew on azole-drug amended media were single-colony isolation streaked on fresh azole-containing media without other antimicrobials added. Several colonies that grew on negative control plates were single-colony isolation streaked and plated to azole-drug amended and non-amended media to identify sensitive isolates for comparison in whole-genome sequencing (WGS) analyses. All collected isolates were stored in 15% glycerol at −80°C.

### MIC assays for antifungal susceptibility testing

Using the Clinical Laboratory Standard Institute broth microdilution method for antifungal susceptibility testing, we tested 160 environmental *A. fumigatus* isolates for sensitivity to the fungicide tebuconazole (TEB; TCI America, Oregon, USA), and the antifungals itraconazole (ITC; Thermo Sci Acros Organics, New Jersey, USA), voriconazole (VOR; Thermo Sci Acros Organics, New Jersey, USA), and posaconazole (POS; Apexbio Technology, Texas, USA). The isolates were grown on complete media slants for 4 days and were then harvested using 3 mL of 0.05% Tween-20 to suspend the hydrophobic spores in solution. The spore suspensions were adjusted to an optical density of 0.09–0.13 at 530 nm using a spectrophotometer, and 100 µL of spore suspension was added to a microtiter plate well containing 100 µL of RPMI 1640 liquid medium (Thermo Sci Gibco, California, USA) and azoles with final concentrations ranging from 0 to 16 µg/mL. The plates were incubated for 48 hours at 37°C and then characterized by the European Committee on Antibiotic Susceptibility Testing (EUCAST) breakpoints (21). Minimum inhibitory concentration (MIC) (21) breakpoints were defined as the lowest concentration where 100% of growth was inhibited. The assay was performed twice on all 160 isolates. For instances where MIC break points differed by greater than one dilution between replicates, the isolates were assayed a third time. For classification of sensitivity or resistance for TEB (>3 µg/mL), ITC (> 2 µg/mL), VOR (> 2 µg/mL), and POS (> 0.25 µg/mL, if = to 0.25 µg/mL consider resistant if ITC is resistant), we used the current recommended clinical breakpoints of antifungal resistance for *A. fumigatus* published by EUCAST 2020 (21, 22).

### DNA extraction and sequencing

Genomic DNA was extracted from *A. fumigatus* isolates using a cetrimonium bromide (CTAB) protocol described previously with some modifications (11, 23). Briefly, cultures were grown overnight in complete medium broth, and approximately 200 mg of mycelium was collected and transferred to 2 mL microcentrifuge tubes containing 150 mg of glass disruption beads and three 3 mm steel beads and lyophilized for 24 hours. Lyophilized cells were disrupted using GenoGrinder 2010 (OPS Diagnostics, Lebanon, NJ) at 1,750 rpm for 30 seconds. To the pulverized tissue, 1 mL of CTAB lysis buffer [100 mM Tris pH 8.0, 10 mM EDTA, 1% CTAB, and 1% β-mercaptoethanol (BME)] was added, and the samples were incubated for 30 minutes at 65°C. After incubation, 300 µL of 5M KAc was added to the samples and incubated on ice for 30 minutes. The supernatant was extracted twice with chloroform, and the aqueous, clear top layer containing DNA was precipitated by adding an equal volume of ice-cold 100% isopropanol, followed by centrifugation. The DNA pellet was washed in 70% EtOH, then 100% EtOH, and air-dried. The resuspended sample was treated with RNase A according to the JGI RNase A DNA cleanup protocol. DNA was checked for quality and quantified using NanoDrop One (Thermo Sci, New Jersey, USA). Library preparation and Illumina NextSeq 2000 P3 sequencing were conducted at the Georgia Genomics and Bioinformatics Core at the University of Georgia, Athens, GA.

### Gene analysis

Whole-genome sequences were assembled for each isolate using SPAdes v3.14.1 with options “–careful” and “–trusted-contigs” (24). Nucleotide BLAST databases were generated for all sequences from SPAdes contig.fasta output using BLAST+ v2.11.0 (25). To investigate genes involved in fungicide resistance, databases were searched by blastn for *A. fumigatus cyp51A* (Afu4g06890), *benA* (Afu1g10910), *cytB* (AfuMt00001), and *sdhB* (Afu5g10370). Blast hits were extracted using BEDtools v2.30.0 (26). Gene analysis was performed using Geneious v2021.0.4.

Genes *MAT1-1-1* (AY898660.1) and *MAT1-2-1* (AFUA_3G06170) indicative of the *MAT1-1* or *MAT1-2* idiomorphs of the mating-type locus (*MAT1*) were used in separate BLAST searches to identify the mating type of each isolate.

To test if Clade 3 was a cryptic species, we used eight housekeeping genes [*act1* (AFUA_6g04740); *efg1* (AFUA_2g02870); *gpdA* (AFUA_5g01970); *histH4* (AFUA_2g13860); ITS region (AFUA_4g02100); *rpb2* (AFUA_7g01920); *sdh1* (AFUA_3g07810); *tef1* (AFUA_1g06390)] that were identified using a BLAST. *Aspergillus fischeri* orthologous and syntenic genes were used as an outgroup [*act1*(NFIA_051290); *efg1* (NFIA_035240); *gpdA* (NFIA_040150); *histH4* (NFIA_089030); ITS region (NR_137479); *rpb2* (NFIA_114650); *sdh1* (NFIA_069350); *tef1* (NFIA_018320)]. As described above, genes were identified and extracted, then analyzed in Geneious v2021.0.4, and aligned using MAFFT version 7.505 with option –auto (27). Alignments were used to construct maximum-likelihood gene trees using IQ-Tree version 2.2.2.6 with options -B 1000 for ultra-bootstraps and -m GTR for the model selection (28).

### Variant calling and phylogenetic analysis

Raw reads were mapped to Af293 (GCF_000002655.1) using BWA v0.7.17 (29). Text pileup outputs were generated for each sequence using SAMtools v1.6 mpileup with option –I to exclude insertions and deletions (30). BCFtools v1.6 call was used to call single nucleotide polymorphisms (SNPs) with options -c to use the original calling method and --ploidy 1 for haploid data (31). Consensus genome sequences in fasta format were extracted from vcf files using seqtk v1.2 (available at https://github.com/lh3/seqtk) with bases with a phred quality score below 40 counted as missing data (N). Because insertions and deletions were removed when mapping reads to the reference, all genome sequences are already mapped to the same coordinates as the reference sequence, and there was, therefore, no need for a multiple alignment step. Variant files (vcf) were used in vcf2allelePlot.pl to test the B-Allele Frequency of all isolates to ensure they were derived from single spores of *A. fumigatus* (32).

MEGA X (33) was used to create a neighbor-joining tree using the Tamura-Nei model with 100 bootstraps for all whole-genome sequences. Neighbor joining is a suitable method for constructing whole-genome trees from SNP data within a frequently recombining species like *A. fumigatus*. Visualization was done using iTOL (34). MEGA X (33) was used to create neighbor-joining gene trees using the Kimura 2-parameter model (35) with 100 bootstraps for all housekeeping genes.

### Population genetic analyses

Principal-component analysis (PCA) was performed using the smartpca program of EIGENSOFT v7.2.1 (36). PCA results were plotted using the ggplot2 package in R version 4.2.1 (37, 38). All vcf files were merged using BCFtools v1.9 and thinned using VCFtools v0.1.16 with options –thin to have one SNP for every 50 nucleotides, --minQ 40 to filter out low-quality SNPs, and –plink to output plink files (ped and map). Plink v1.9b was used with the ped and map files as input and option --make-bed to output a bed file to be used in ADMIXTURE (39). To identify ancestry and admixed strains, ADMIXTURE v 1.3 was used in five independent runs (1, 12,345, 33,333, 54,321, and 98,765) with 2–20 clusters (K) (40). Resulting Q files were used in the Distruct for many K’s features of CLUMPAK to align cluster labels across different models (41). CLUMPAK results were added to R version 3.5.0 to be plotted (37). Cross-validation values for all five runs K2-20 can be found in Fig. S4. The neighbor-joining tree, PCA, critical values (BIC), and loglikelihood values of each K were used to identify the most likely number of clusters (K).

## RESULTS

To better understand the prevalence of azole-resistant *A. fumigatus* in U.S. agricultural environments, we collected soil, plant debris, and compost from 29 agricultural sites in five eastern states (New York, Pennsylvania, Virginia, South Carolina, and Georgia) and from 23 agricultural sites in three western states (Washington, Oregon, and California). Crops cultivated on our sites included grains, fruits, nuts, and ornamentals (Table 1). With the exception of Georgia, no surveys to detect azole-resistant *A. fumigatus* in agricultural settings have been reported for these eight states. We plated samples onto media containing either no azole, the agricultural fungicide TEB, or the clinical antifungal drug ITC and isolated a total of 727 isolates of *A. fumigatus*. Following this preliminary selection, we determined the MIC for the clinical antifungal agents ITC, VOR, and POS by broth dilution assays for 202 isolates representing all sites and including azole-sensitive and -resistant isolates. Of these isolates, 44 were sensitive to all three azoles tested, 24 were resistant to ITC, 25 were resistant to VOR, and 109 were resistant to POS, with 34 of those showing resistance to more than one clinical azole drug (pan-azole resistant) based on EUCAST 2020 cutoffs (Table 1) (21, 22). We performed WGS on 135 isolates representing the range of collection sites and azole sensitivities.

**TABLE 1:** Sampling of *A. fumigatus* strains from agricultural sites

Crop/substrate sampled	Location(s) sampled	Sampling date(s) MM/DD/YY (no. sites)	Isolates collected, no.					Whole-genome sequencing strains (*azole-resistant)^*b*^
			Total # isolates obtained (# tested)	MIC				
				S	Resistant^*a*^			
					ITC	VOR	POS	
Wheat	Cayuga Co., NY Benton Co., OR Marion/Linn Co., OR Suffolk, VA	11/07/18 (4) 05/13/19 (2) 05/15/19 (1) 08/29/19 (1)	89 (26) 4 (2) 0 (0) 8 (1)	7 2 0 1	1 0 0 0	0 0 0 0	19 0 0 0	1,001*, 1,003*, 1,006*, 1,008*, 1,010*, 1,011*, 1,014*, 1,015*, 1,018*, 1,023, 1,030, 1,054*, 1,057*, 1,060, 1,068, 1,075*, 1,082*, 1,083*, 1,085, 1,432*, 1,433*, 1,434*, 1,435*, and 1,592
Apple	Adams Co., PA	11/08/18 (4)	79 (10)	4	0	0	6	1,086*, 1,090, 1,092*, 1,130, 1,141, 1,175*, 1,177, 1,178*, and 1,196*
Peach	Adams Co., PA Anderson Co., SC Oconee Co., SC	11/08/18 (1) 09/09/19 (1) 09/09/19 (1)	25 (6) 10 (2) 3 (3)	3 1 2	0 1 0	0 1 0	3 1 1	1,107, 1,110*, 1,113, 1,114*, 1,125, 1,126*, 1,649*, 1,650, and 1,659
Grapes	Ontario Co., NY Benton Co., WA Fresno Co., CA Marion Co., OR Napa Co., CA	11/29/18 (1) 12/02/18 (1) 12/03/18 (1) 07/15/19 (1) 07/18/19 (1) 07/26/19 (3) 08/01/19 (1) 08/09/19 (3) 08/26/19 (2)	8 (1) 1 (1) 3 (1) 5 (1) 5 (1) 25 (7) 8 (1) 64 (14) 38 (23)	1 1 1 1 1 1 1 1 3	0 0 0 0 0 4 0 2 0	0 0 0 0 0 4 0 3 0	0 0 0 0 0 6 0 13 20	1,145, 1,150, 1,153, 1,154, 1,478, 1,482, 1,490, 1,500*, 1,502, 1,504*, 1,508, 1,510, 1,515*, 1,519*, 1,520*, 1,525*, 1,528*, 1,529*, 1,530, 1,535*, 1,546*, 1,547*, 1,548*, 1,555*, 1,560*, 1,562, 1,570*, 1,572*, 1,576*, 1,577*, 1,580*, 1,615, 1,619*, and 1,638*
Wood chip compost	Clarke Co., GA	01/07/19 (1)	39 (4)	3	0	0	1	1,205, 1,209*, 1,215, and 1,227
Horse manure compost	Clarke Co., GA	01/07/19 (1)	76 (9)	0	4	0	9	1,361*, 1,368*, 1,376*, 1,392*, 1,416*, 1,420*, 1,426*, 1,428*, and 1,430*
Longwood compost	Clarke Co., GA	01/07/19 (1)	19 (9)	0	0	0	9	1,436*, 1,438*, 1,442*, 1,444*, 1,447*, 1,448*, 1,449*, 1,453*, and 1,454*
Mixed brassicas	Clarke Co., GA	01/07/19 (2)	0 (0)	0	0	0	0	
Mixed herbs	Clarke Co., GA	01/07/19 (2)	115 (8)	4	0	0	4	1,245, 1,267, 1,280*, 1,305, 1,306*, 1,319*, 1,349, and 1,350*
Tulips	Clackamas Co., OR^*c*^ Skagit Co., WA Pierce Co., WA	05/13/19 (1) 10/07/19 (2) 10/14/19 (4) 10/14/19 (2)	24 (16) 8 (1) 32 (4) 17 (0)	1 1 2 0	11 0 1 0	15 0 2 0	15 0 2 0	1,472*, 1,473, 1,457*, 1,458*, 1,670*, 1,673, 1,683, 1,685*, 1,700, 1,703*, 1,718*, and 1,719
Hemp	Clackamas Co., OR^*c*^ Barnwell Co., SC	05/13/19 (1) 06/20/19 (1)	24 (16) 0 (0)	1 0	11 0	15 0	15 0	1,472*, 1,473, 1,457*, 1,458*, 1,459*, 1,460*, 1,461, 1,465*, 1,467*, 1,468*, 1,469*, 1,470*, 1,664*, 1,665*, 1,666*, 1,667*, 1,668*, and 1,669*
Watermelon	Barnwell Co., SC Suffolk, VA	06/20/19 (1) 08/29/19 (1)	0 (0) 6 (1)	0 1	0 0	0 0	0 0	1,584
Cantaloupe	Barnwell Co., SC	06/20/19 (1)	0 (0)	0	0	0	0	
Peanut	Suffolk, VA	08/29/19 (1)	8 (1)	1	0	0	0	1,596
Corn and soy	Suffolk, VA	08/29/19 (1)	8 (1)	1	0	0	0	1,606
Total		52 sites	727 (202)	44	24	25	109	135

^*a*^ # tested indicates isolates for which MIC was determined. Some isolates were resistant to more than one azole. For more detail see Table S2.

^*b*^ Asterisks indicate azole-resistant isolates based on minimum inhibitory concentration (MIC). Bold numbers indicate isolates resistant to more than one azole based on MIC (pan-azole-resistant isolates).

^*c*^ These isolates come from a field cultivated with both hemp and tulips.

**Fig 1:**
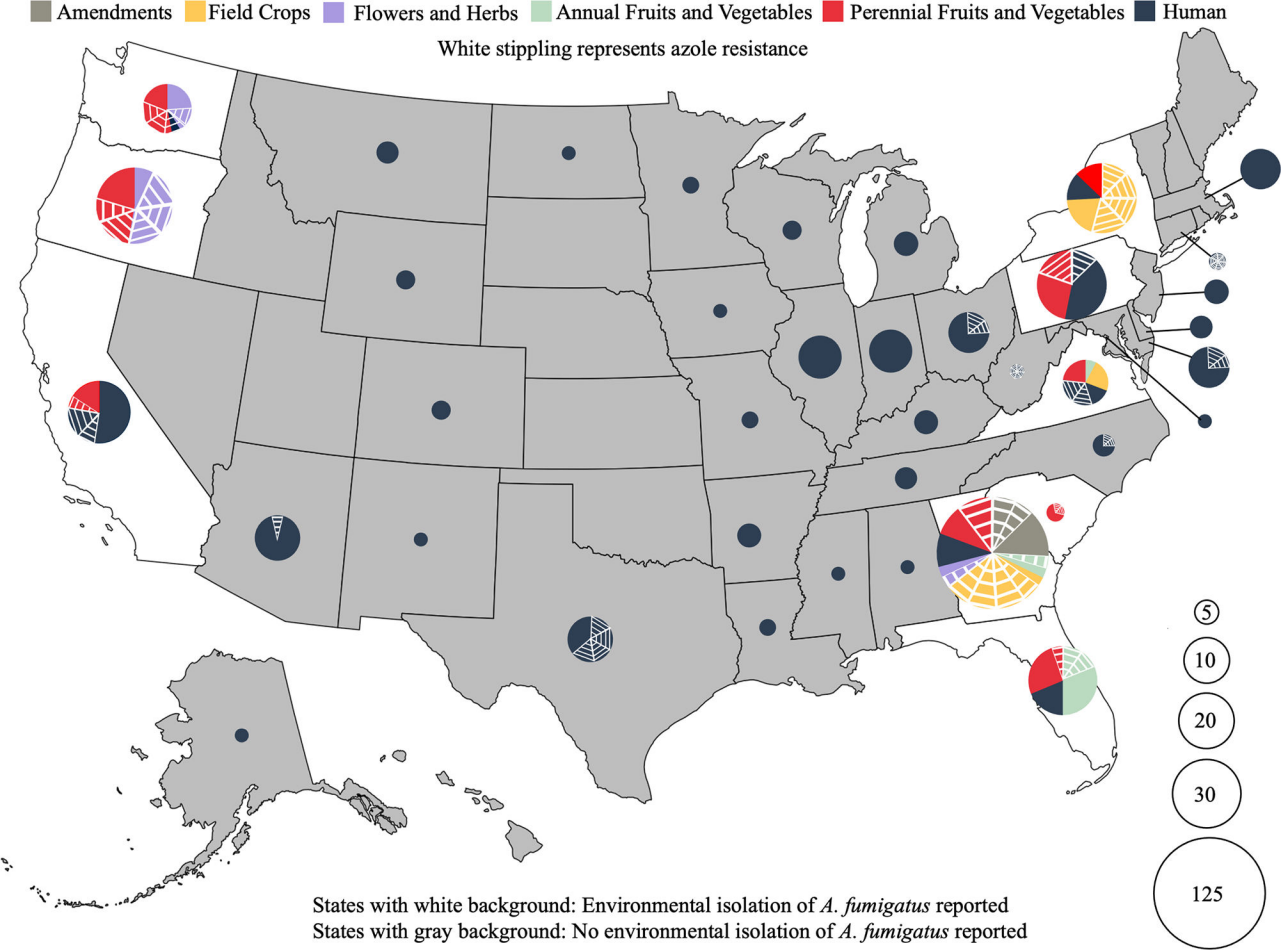
Locations of all *A. fumigatus* isolated from clinical and environmental settings in the U.S. as of June 2023. States with gray background have no reported environmental sampling of *A. fumigatus*. The size of the pie charts represents the number of isolates reported in each state. Sampling intensity varied widely, so the isolate number does not accurately reflect the relative abundance of *A. fumigatus* in each state. Pie chart colors represent sample types (gray: soil amendments; yellow: field crops; purple: flowers and herbs; green: annual fruits and vegetables; red: perennial fruits and vegetables; dark blue: human clinical). White stripes on pie charts denote azole-resistant isolates. It should be noted that samples in the field crop category in Georgia are primarily from a single peanut cull pile in which multiple azole-resistant isolates were detected. Based on the current study and publicly available data from previous studies as shown in Table S1 (11, 13, 14, 20, 42–50). The map was created using the Cran-R project (https://cran.r-project.org/web/packages/usmap/index.html).

Previous studies reported on clinical *A. fumigatus* isolated from healthcare settings in 37 states (13) and environmental *A. fumigatus* isolated from agricultural settings in Florida and Georgia (11, 20). We placed our new isolates and data for 295 publicly available isolates on a map allowing visualization of the distribution of all clinical and environmental *A. fumigatus* so far reported in the U.S. (Fig. 1; Table S1). It is impossible to determine the actual frequency of azole-resistant *A. fumigatus* in individual states or associated with particular crops or substrates because sampling intensity and isolate screening and selection have varied widely; however, it is clear that azole-resistant *A. fumigatus* is widespread geographically and across crops and substrates. Pan-azole-resistant isolates were prevalent among samples from grape, compost, tulips, and hemp (Table 1). *A. fumigatus* isolates have been reported from 38 states. Of these isolates, 241 were collected from environmental settings in 9 states and 189 from clinical settings in 37 states; 263 were sensitive to clinical azole drugs, 101 to a single azole, and 66 to more than one azole (pan-azole resistant). Including the present study, agricultural sites in only nine states have been sampled; azole-resistant *A. fumigatus* was not detected in agricultural samples from Virginia but was detected in agricultural samples from the other eight states surveyed (Fig. 1; Table S1).

**Fig 2:**
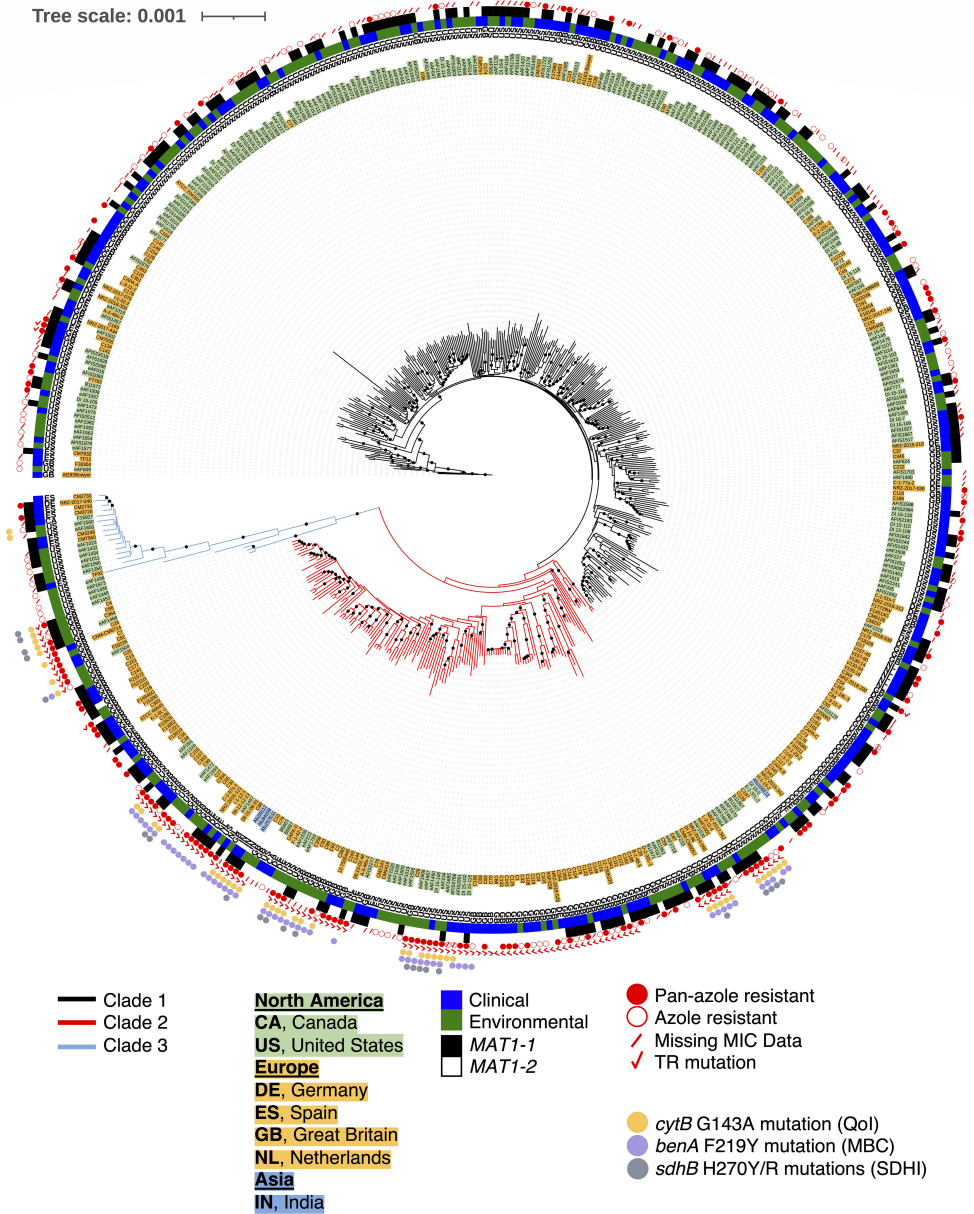
Neighbor-joining tree of 480 subsampled environmental and clinical isolates of *A. fumigatus*. Whole-genome sequences from agricultural sites on the east and west coasts of the United States (eAF1XXX) were analyzed along with publicly available data (Table S1). Af293 was used as the reference genome and root of the tree. Bootstrap values greater than 95% are indicated by a dot on the branch. Clade 1 branches are black; Clade 2 branches are red; Clade 3 branches are blue. Country of origin is listed next to each isolate according to their two-letter designation (CA, Canada; DE, Germany; ES, Spain; GB, Great Britain; IN, India; NL, Netherlands; US, United States). North American isolates are shown with a green background, European with gold, and Asian with blue. Green and blue bars indicate environmental and clinical isolates, respectively. Black and white bars represent mating types *MAT1-1* and *MAT1-2*, respectively. Open red circles indicate azole-resistant isolates. Solid red circles indicate pan-azole-resistant isolates (i.e., isolates resistant to at least two different clinical azoles based on MIC testing). Red slash marks represent isolates without MIC test data for either itraconazole, voriconazole, or posaconazole. Red check marks indicate isolates with TR mutations. Yellow circles indicate the *cytB* G143A mutation conferring resistance to QoI fungicides (51). Purple circles indicate the *benA* F219Y mutation conferring resistance to MBC fungicides (52). Gray circles indicate the *sdhB* H270Y/R mutations conferring resistance to SDHI fungicides (53).

Several studies showed that worldwide *A. fumigatus* populations fell into two clusters (Clade A and Clade B) with most TR_34_/L98H and TR_46_/Y121F/T289A-based pan-azole resistance in Clade A (12–14). A recent pan-genome study including more isolates from around the world showed that *A. fumigatus* populations fell into three clusters: Clade 1 contains fewer azole-resistant strains and appears to be the same as the previously identified Clade B; Clade 2 contains many TR-based pan-azole isolates and appears to be the same as Clade A. The small Clade 3 has no azole resistance and is made up of 14 clinical isolates from Europe, the U.S., Canada, and a single environmental isolate from Peru (16). To determine the relationships of U.S. agricultural isolates from the current study to worldwide *A. fumigatus* clinical and agricultural populations, we performed whole-genome sequencing on 135 and used the resulting sequences, along with 594 publicly available whole-genome sequences to construct a neighbor-joining tree of 729 total sequences (Fig. S1; Table S2). To better visualize the tree, 482 representative isolates were chosen for subsampling (Fig. 2; Table S2). In addition to analyzing azole-resistance mutations in *cyp51A*, we analyzed genes responsible for resistance to agricultural fungicides [*benA* for benzimidazole (MBC) class, *cytB* for quinone outside inhibitor (QoIs), and *sdhB* for succinate dehydrogenase inhibitor (SDHI) class] and mating-type (*MAT1-1-1*, *MAT1-2-1*; AY898660.1, AFUA_3G06170).

Our phylogenetic analysis of 729 worldwide isolates was consistent with three clades with the branch leading to the small Clade 3 showing 100% bootstrap support (Fig. 2; Fig. S1). *MAT1-1* and *MAT1-2* isolates were present in almost equal proportions throughout the tree (354/729 and 375/729, respectively), and there was no association with clade designation (Fig. 2; Fig. S1). Based on sequence data and single mutations known to be associated with fungicide resistance phenotypes in *A. fumigatus* (11, 53, 54), we classified isolates as sensitive or resistant to the agricultural fungicides MBC, QoI, and SDHI (Table S2). We used MIC data for ITR, VOR, and POS to determine if isolates were sensitive and resistant to a single clinical azole (azole resistant) or to multiple clinical azoles (pan-azole resistant). Because MICs for all three clinical azoles were not reported for all publicly available strains, the actual number of pan-azole-resistant isolates might be higher than our data suggest. Clade 1 was the largest containing 463/729 total isolates. A majority of Clade 1 isolates were azole-sensitive (292/463) and of the 171 azole-resistant isolates, 80/171 were resistant to more than one azole, and only five had TR mutations in the *cyp51A* promoter. No Clade 1 isolates had the known mutations associated with resistance to MBC, QoI, or SDHI agricultural fungicides. Clade 2 was the next largest group containing 208/729 isolates. Most Clade 2 isolates were azole-resistant (154/208) with 103 being pan-azole resistant and 128 carrying a TR mutation in the *cyp51A* promoter. Seventy-nine Clade 2 isolates had the known mutations associated with resistance to MBC, QoI, or SDHI agricultural fungicides with 53/79 indicating resistance to more than one agricultural fungicide (multi-fungicide resistant). Interestingly all fungicide-resistant isolates in Clade 2 also contained a TR mutation (79/79). Clade 3 was the smallest (39/729) and contained azole-sensitive (17/39), azole-resistant (20/39), pan-azole-resistant (2/39), and fungicide-resistant isolates (4/39), though no TR or multi-fungicide resistance mutations were present.

The presence of many TR mutations in Clade 2 and a few in Clade 1 (C109, C141, C134, and NRZ-2017–214) is similar to the findings of previous studies conducted with different isolates (Fig. S1) (14, 42). Of the azole-resistant isolates in Clade 1 (Fig. S1), roughly 10% had no *cyp51A* mutations, and 64% had *cyp51A* mutations that have not been shown to cause azole resistance (A9T, Y46F, V172M, T248N, E255D, K427E, and Wild Type (WT); Table S1). Multi-fungicide resistance was only found in Clade 2 and was mostly associated with isolates with a TR mutation in *cyp51A* (Table 2).

**TABLE 2:** Mutations associated with multi-fungicide resistance in azole-resistant *A. fumigatus*

Isolate	Source	Clade	*cyp51A*-azoles	*cytB*- QoI^*a*^	*benA*-MBC^*b*^	*sdhB*-SDHI^*c*^	Reference (DOI)
Af293 - Bowyer	Clinic, UK	1	WT	WT	WT	WT	10.3390/genes9070363
eAF1438	Environment, USA	3	T248N/E255D/K427E	WT	WT	WT	This study
eAF1650	Environment, USA	3	T248N/E255D	G143A	WT	WT	This study
AFIS2001	Clinic, USA	2	TR34/Y46F/L98H/V172M/T248N/E255D/K427E	G143A	F219Y	WT	10.1128/mBio.01803-21
Afu 1042/09	Clinic, India	2	TR34/Y46F/L98H/V172M/T248N/E255D/K427E	G143A	F219Y	WT	10.1128/mBio.00536-15
Afu 124/E11	Environment, India	2	TR34/Y46F/L98H/V172M/T248N/E255D/K427E	G143A	F219Y	WT	10.1128/mBio.00536-15
C4	Environment, Wales	2	TR34/Y46F/L98H/V172M/T248N/E255D/K427E	G143A	WT	H270Y	10.1038 /s41564-022-01091-2
C10	Environment, Wales	2	TR34/Y46F/L98H/V172M/T248N/E255D/K427E	G143A	F219Y	H270R	10.1038 /s41564-022-01091-2
C13	Environment, Wales	2	TR34/Y46F/L98H/V172M/T248N/E255D/K427E	G143A	F219Y	H270Y	10.1038 /s41564-022-01091-2
C14	Environment, Wales	2	TR34/Y46F/L98H/V172M/T248N/E255D/K427E	G143A	F219Y	WT	10.1038 /s41564-022-01091-2
C28	Environment, Wales	2	TR34/Y46F/L98H/V172M/T248N/E255D/K427E	G143A	WT	H270Y	10.1038 /s41564-022-01091-2
C86	Environment, Ireland	2	TR34/Y46F/L98H/V172M/T248N/E255D/K427E	G143A	WT	H270Y	10.1038 /s41564-022-01091-2
C364	Environment, Scotland	2	TR34/Y46F/L98H/V172M/T248N/E255D/K427E	G143A	WT	WT	10.1038 /s41564-022-01091-2
eAF1468	Environment, USA	2	TR34/Y46F/L98H/V172M/T248N/E255D/K427E	G143A	WT	H270Y	This study
eAF1472	Environment, USA	2	TR34/Y46F/L98H/V172M/T248N/E255D/K427E	G143A	F219Y	H270Y	This study
eAF1570	Environment, USA	2	TR34/Y46F/L98H/V172M/T248N/E255D/K427E	G143A	WT	H270Y	This study
eAF1668	Environment, USA	2	TR34/Y46F/L98H/V172M/T248N/E255D/K427E	G143A	F219Y	H270Y	This study
eAF1667	Environment, USA	2	TR34/Y46F/L98H/V172M/T248N/E255D/K427E	WT	F219Y	H270Y	This study
eAF1535	Environment, USA	2	TR46/Y46F/Y121F/V172M/T248N/T298A/K427E	G143A	F219Y	H270Y	This study
DI_15–106	Clinic, USA	2	TR46/Y121F/V172M/T248N/T298A/E255D/K427E	G143A	F219Y	H270Y	10.1128/JCM.02478-15
C155	Clinic, England	2	TR34/Y46F/L98H/V172M/T248N/E255D/K427E/G448S	G143A	F219Y	WT	10.1038 /s41564-022-01091-2
C89	Environment, Ireland	2	TR46/Y46F/Y121F/V172M/T248N/E255D/T289A/K427E	G143A	F219Y	WT	10.1038 /s41564-022-01091-2
B11982	Environment, USA	2	TR34/Y46F/L98H/V172M/T248N/E255D/S297T/K427E/F495I	G143A	F219Y	WT	10.1128/mBio.01803-21
DI_15–96	Clinic, USA	2	TR46/Y46F/Y121F/V172M/T248N/E255D/T298A/K427E	G143A	F219Y	WT	10.1128/JCM.02478-15
eAF222	Environment, USA	2	TR46/Y46F/Y121F/V172M/T248N/E255D/T298A/K427E	G143A	F219Y	H270Y	10.1093/g3journal/jkab427
eAF234	Environment, USA	2	TR46/Y46F/Y121F/V172M/T248N/E255D/T298A/K427E	G143A	F219Y	WT	10.1093/g3journal/jkab427
eAF513	Environment, USA	2	TR46/Y46F/Y121F/V172M/T248N/E255D/T298A/K427E	G143A	F219Y	WT	10.1093/g3journal/jkab427
eAF1458	Environment, USA	2	TR46/Y46F/Y121F/V172M/T248N/E255D/T298A/K427E	G143A	F219Y	H270Y	This study
eAF1685	Environment, USA	2	TR46/Y46F/Y121F/V172M/T248N/E255D/T298A/K427E	G143A	F219Y	H270R	This study
eAF1703	Environment, USA	2	TR46/Y46F/Y121F/V172M/T248N/E255D/T298A/K427E	G143A	F219Y	WT	This study
C93	Environment, Ireland	2	TR46/Y46F/Y121F/V172M/T248N/E255D/T289A/K427E/G448S	G143A	F219Y	H270Y	10.1038/s41564-022-01091-2

^*a*^ Mutation G143A in *cytB* causes resistance to QoIs (55).

^*b*^ Mutation F219Y in *benA* causes resistance to MBCs (56).

^*c*^ Mutation H270Y/R in *sdhB* causes resistance to SDHIs (57).

**Fig 3:**
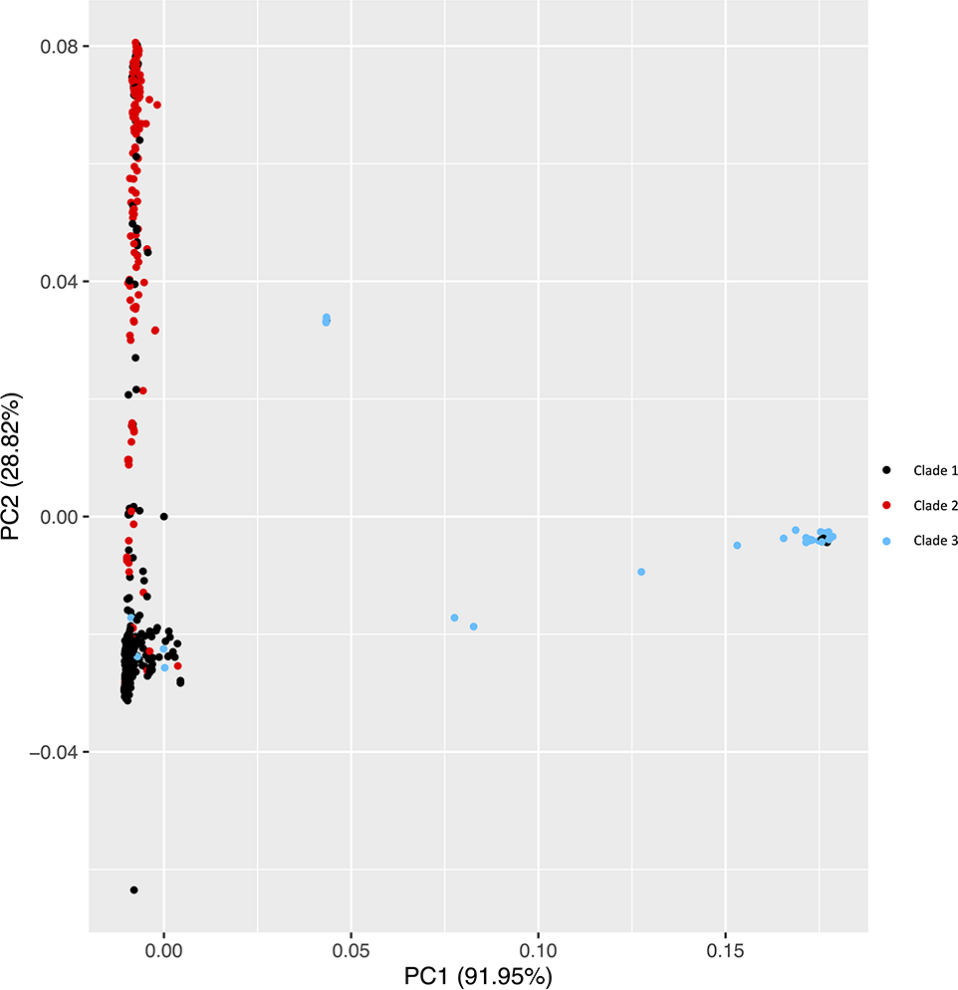
Principal components (PC) 1 and 2 plot mostly separate the three clades. Clade 1 is represented by black dots. Clade 2 is represented by red dots. Clade 3 is represented by blue dots. PC 1 represents 41.59% of data along the x-axis. PC 2 represents 13.04% of data on the y-axis.

To further investigate population structure, all 729 isolates were included in a PCA. Isolates congregated in three main clusters supporting a three-clade structure with some presumed recombinant isolates intermixed among all clades (Fig. 3).

**Fig 4:**
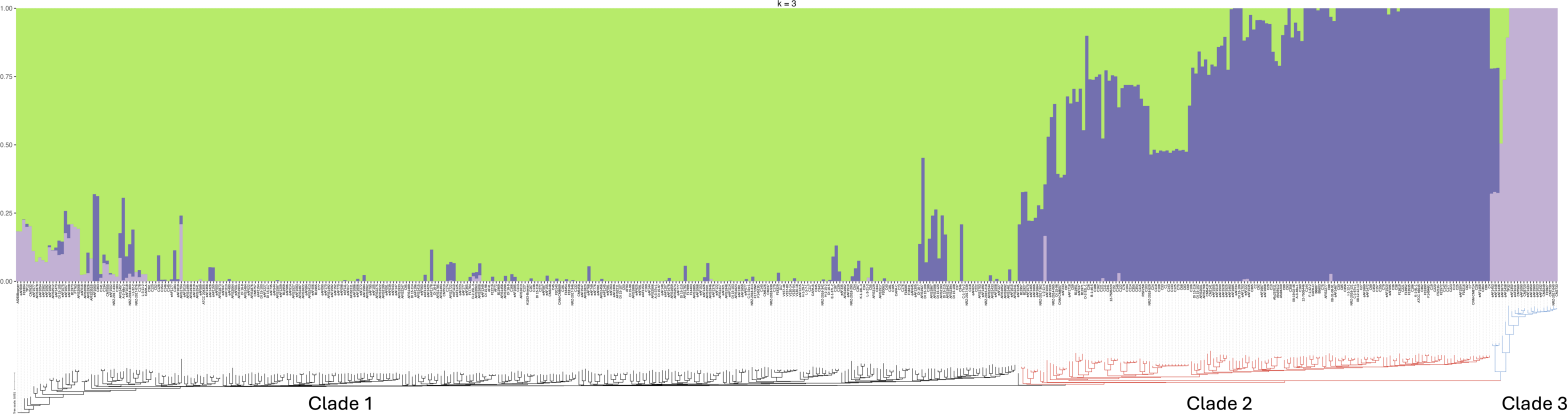
ADMIXTURE analysis supports three populations of *A. fumigatus* with recombination among clades. Clade 1 is mostly aligned with the green cluster. Clade 2 is mostly aligned with the dark purple cluster. Clade 3 mostly aligns with the light purple cluster. Some isolates in every clade show admixture with the green and other clusters. Numbers represent clade number. Y-axis represents ancestry. A rectangular version of the neighbor-joining subsample tree (Fig. 2) is on the x-axis. Black branches represent Clade 1. Red branches represent Clade 2. Blue branches represent Clade 3. For cross-validation plots see Fig. S3.

To further investigate presumed recombination between clades, we used our 480 isolate subsample group of *A. fumigatus* U.S. agricultural and worldwide isolates to conduct ADMIXTURE analysis. ADMIXTURE was run with five different seeds for *K* values 2 through 20, and the best log likelihood *K* values were analyzed (Fig. S2 and S3). K3 best fits the data and supports three clusters that mostly align with the three clades but also show gene flow between clades (Fig. 4).

**Fig 5:**
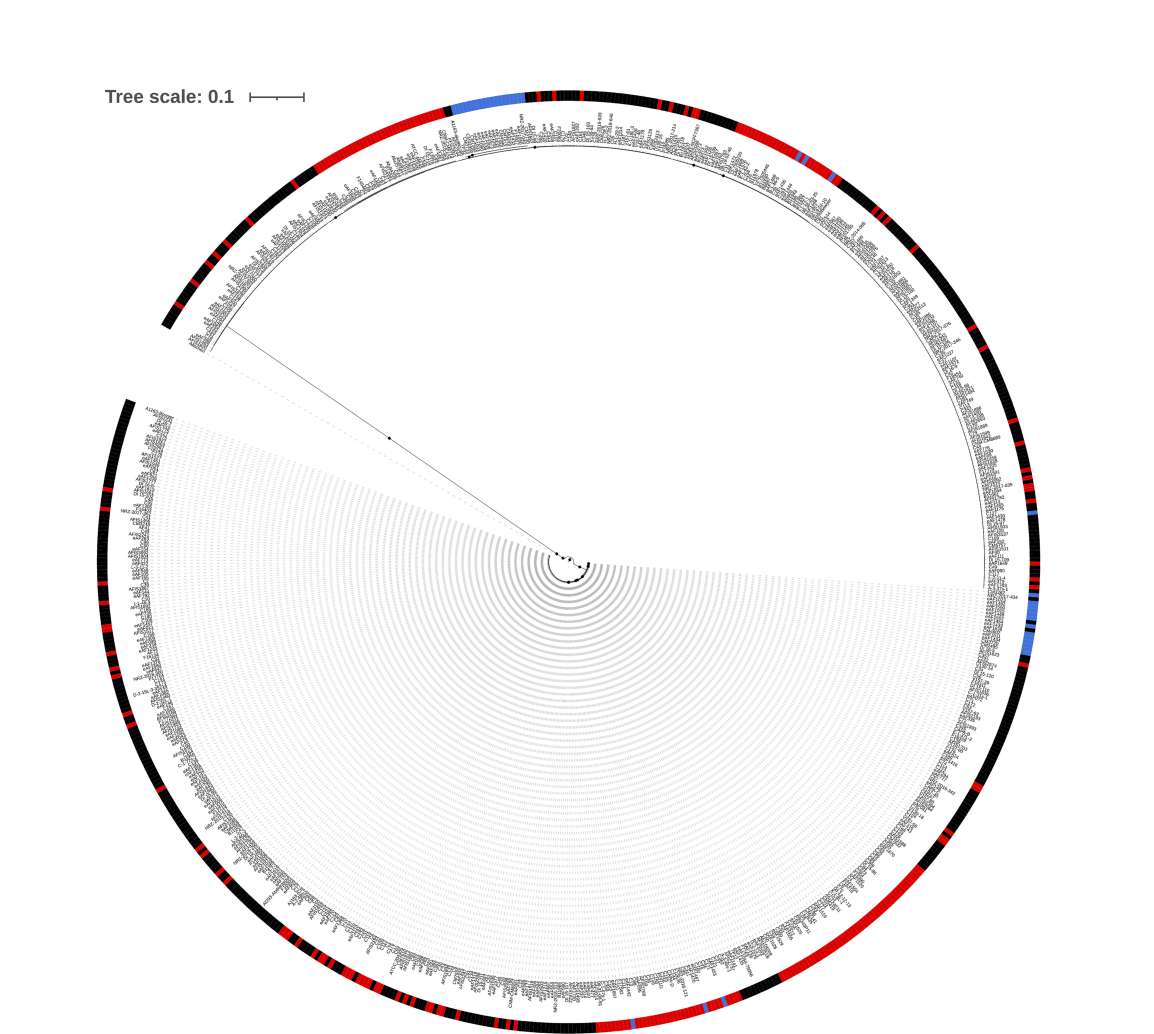
Gene trees of housekeeping genes show that Clade 3 is not monophyletic. Gene trees of *act1* (shown) and seven other housekeeping genes (Fig. S4) from 729 *A*. *fumigatus* isolates and *A. fischeri* as outgroup show that Clade 3 is not monophyletic, supporting the view that these isolates are *A. fumigatus* and not a cryptic species. Circles on branches represent bootstraps over 90. Black squares represent isolates from Clade 1. Red squares represent isolates from Clade 2. Blue squares represent isolates from Clade 3.

Our phylogenetic, ADMIXTURE, and PCA analyses all suggested three populations with the small Clade 3 being the most diverged from the other two. To determine whether Clade 3 might be a cryptic species distinct from *A. fumigatus*, we constructed gene trees for eight different housekeeping genes (*act1*, *efg1*, *gapdh*, *histH4*, *ITS*, *rpb2*, *sdh1*, and *tef1*) from all 729 isolates with *A. fischeri* as an outgroup (Fig. 5; Fig. S4). In no case did Clade 3 form a monophyletic group that diverged from Clades 1 and 2 as would be expected if it was a different species nor did Clade 3 group with *A. fischeri*. In six of the eight trees, Clade 3 isolates were mostly clustered together but not monophyletic, and Clades 1 and 2 isolates were interspersed suggesting Clade 3 is rapidly diverging from Clades 1 and 2 (Fig. S4). In two of the eight trees, *gapdh* and ITS isolates from all three clades were interspersed showing less divergence among clades for these specific loci. The absence of monophyly for the gene trees for clade 3 isolates shows current or historically recent gene flow and thus indicates that it is not a distinct species.

## DISCUSSION

### Azole-resistant *A. fumigatus* is widespread in the U.S.

Before the current study, *A. fumigatus* isolates from clinical sources had been reported in 37 states and from environmental sources in two states [Georgia and Florida (11, 20, 55); Fig. 1]. We surveyed agricultural sources in seven additional states including four on the eastern U.S. coast and three on the western U.S. coast. We combined our data with publicly available data to get an overview of *A. fumigatus* reported from U.S. clinical and environmental sources. Sampling intensity has varied widely, so we could not determine the true prevalence of azole-resistant *A. fumigatus* in each state or associated with different crops. However, it is clear that azole-resistant *A. fumigatus* is widespread in the U.S. We found it in agricultural environments on both coasts where a variety of crop types have been cultivated. Interestingly, we found high levels of TR-based azole resistance and multi-fungicide resistance in isolates from tulip farms on the west coast that also grow hemp (Table S1). Most tulips in the U.S. are grown from bulbs imported from the Netherlands, where TR-based azole resistance was first reported and is thought to have originated (56, 57).

### Our data support three clades of *A. fumigatus* worldwide

Previous work from multiple labs showed that worldwide *A. fumigatus* populations were not structured by geography (43, 58, 59). Most recent work using clinical and environmental isolates suggested that *A. fumigatus* populations fell into two major clades with most TR-based pan-azole-resistance in Clade A (11–13). A notable exception is a pan-genome analysis which showed seven genetic clusters of *A. fumigatus*, though this analysis also found close clustering of TR-based azole resistance and no strong geographic structure (42). A recent pan-genome analysis with increased sampling identified three clades: a large group with few azole-resistant isolates called Clade 1 by the authors (equivalent to Clade B in earlier work), a somewhat smaller group of isolates that included most isolates with TR-based pan-azole resistance called Clade 2 by the authors (equivalent to Clade A in earlier work), and a third clade with the fewest number of isolates and no azole resistance called Clade 3 by the authors (16). In the current study, our phylogenetic, PCA, and ADMIXTURE analyses of 729 U.S. and worldwide isolates support three clades: Clade 1 with 463 isolates (equivalent to Clade B in previous work), Clade 2 with 208 isolates (equivalent to Clade A in previous work), and the small Clade 3 with 39 isolates (Fig. 2 to 4). To ensure that Clade 3 was not a cryptic species, we performed phylogenetic analyses with eight housekeeping genes using *A. fischeri* as an outgroup. In all cases, the tree topology was consistent with polyphyly for Clade 3, not the monophyly which would be expected for a cryptic species. The clustering of Clade 3 isolates among trees supported the presence of a third clade of *A. fumigatus* but not a cryptic species (Fig. 5; Fig. S4). Based on phylogenetic analysis of whole-genome sequence (Fig. 2; Fig. S1) and individual housekeeping genes (Fig. 5; Fig. S4), Clade 3 appears to be recently diverged from Clades 1 and 2.

### TR-based pan-azole resistance and multi-fungicide resistance are strongly associated

We found that 128 isolates that contained a TR mutation in the *cyp51A* promoter were in Clade 2, five were in Clade 1, and none were in Clade 3, similar to previous reports which included fewer U.S. isolates (16). Interestingly, none of the 83 isolates resistant to the agricultural fungicides MBC, QoI, or SDH were in Clade 1 with the remaining four in Clade 3. A total of 79 fungicide-resistant isolates were in Clade 2, and all 79 also carried either the TR_34_ or the TR_46_ azole-resistance alleles of *cyp51A* (Fig. S1; Table S2). This pattern suggests that the genetic background of Clade 2 isolates allows more rapid adaptation to environmental stress.

Though we found no TR-based resistance in Clade 3, we did find two pan-azole-resistant isolates that did not carry a TR allele and 20 isolates that were resistant to the single-azole POS. Generally, our study found more POS resistance (and hence non-pan-azole resistance) than reported in other studies. We think this is because MICs are less often determined for POS than for ITC and VOR. In our cumulative data set, 35% of isolates do not have MICs reported for POS, while only 14% lack MICs for ITC and VOR (Table S2).

### U.S. populations show signatures of recombination

Previous work from others found evidence of very high recombination rates in *A. fumigatus* with recombination frequently occurring between clades (16, 60). Our isolates also showed signatures of recombination between clades in PCA and ADMIXTURE analysis (Fig. 3 and 4).

### Conclusion

Our study expands environmental sampling of *A. fumigatus* in the U.S. We show that azole-resistant and multi-fungicide-resistant isolates are found in agricultural settings in both the east and west coast regions of the country in areas where a variety of crops have been cultivated. We show support for three clades of *A. fumigatus*, consistent with a recent study that included many fewer U.S. isolates (Fig. 3 and 4) (16). We found that while the small Clade 3 has no TR-based azole resistance, it contains isolates with resistance to single and multiple azoles and to the QoI fungicides. Interestingly, the remaining 79 isolates that were resistant to agricultural fungicides in the MBC, QoI, or SDHI classes were in Clade 2, and all also carried a TR allele of *cyp51A* associated with pan-azole resistance. U.S. isolates also showed high levels of recombination raising the worrying prospect that resistance to clinical azoles will continue to spread in the U.S.

## Data Availability

Sequences were deposited in NCBI under project PRJNA991533.

## SUPPLEMENTAL MATERIAL

Fig. S1.Neighbor-joining tree of 729 environmental and clinical isolates of Aspergillus fumigatus aem.01782-23-s0001.tif Whole-genome sequences from agricultural sites in the east and west coast regions of the United States (eAF1XXX) were analyzed with publicly available data. Af293 was used as the reference genome and root of the tree. Bootstrap values are indicated on each branch. Clade 1 branches are black; Clade 2 branches are red; Clade 3 branches are blue. Country of origin is listed next to each isolate according to their two-letter designation (CA, Canada; DE, Germany; ES, Spain; GB, Great Britain; IN, India; NL, Netherlands; US, United States). Green and blue bars indicate environmental and clinical isolates, respectively. Black and white bars represent mating type MAT1-1 and MAT1-2, respectively. Open red circles indicate azole-resistant isolates. Solid red circles indicate pan-azole-resistant isolates (i.e., isolates resistant to at least two different clinical azoles based on MIC testing). Red slash marks represent isolates without MIC test data for either itraconazole, voriconazole, or posaconazole. Red check marks indicate isolates with TR mutations. Yellow circles indicate the cytB G143A mutation conferring resistance to QoI fungicides. Purple circles indicate the benA F219Y mutation conferring resistance to MBC fungicides. Gray circles indicate the sdhB H270Y/R mutations conferring resistance to SDHI fungicides.

Figure S2. ADMIXTURE analysis of K2-20 aem.01782-23-s0002.tif Numbers within plots represent populations. Y-axis represents ancestry. A rectangular version of the neighbor-joining subsample tree (Figure 2) is on the x-axis. Black branches represent Clade 1. Red branches represent Clade 2. Blue branches represent Clade 3. Cross validation values are shown in Figure S3.

Figure S3. Cross validation aem.01782-23-s0003.tif Cross validation values for ADMIXTURE analysis shown in Figure S2.

Figure S4. Gene trees of housekeeping genes and ITS region aem.01782-23-s0004.tif Gene trees with 729 isolates were constructed for act1, efg1, gapdh1, histh4, the ITS region, rpb2, sdh1, and tef1. Circle size on branches is a proxy for bootstrap values. Black squares represent isolates from Clade 1. Red squares represent isolates from Clade 2. Blue squares represent isolates from Clade 3.

Supplemental material legends aem.01782-23-s0005.docx Legends of supplemental figures and tables.

Table S1. Metadata for US isolates aem.01782-23-s0006.xlsx Metadata for 431 U.S. isolates used in map.

Table S2. Metadata of all isolates aem.01782-23-s0007.xlsx Metadata for all 729 worldwide isolates used in this study.

ASM does not own the copyrights to Supplemental Material that may be linked to, or accessed through, an article. The authors have granted ASM a non-exclusive, world-wide license to publish the Supplemental Material files. Please contact the corresponding author directly for reuse.
