# Supplementary material for: Pan-azole- and multi-fungicide-resistant Aspergillus fumigatus is widespread in the United States
Source: Appl Environ Microbiol. 2024 Apr 1;90(4):e01782-23. doi: 10.1128/aem.01782-23 (PMC11022549; doi:10.1128/aem.01782-23)
Supplement: Supplemental material legends — Legends of supplemental figures and tables. [file aem.01782-23-s0005.docx]

**Supplemental Materials Legends**

**Figure S1. Neighbor-joining tree of 729 environmental and clinical isolates of *Aspergillus fumigatus****.* Whole genome sequences from agricultural sites in the east and west coast regions of the United States (eAF1XXX) were analyzed with publicly available data. Af293 was used as the reference genome and root of the tree. Bootstrap values are indicated on each branch. Clade 1 branches are black; Clade 2 branches are red; Clade 3 branches are blue. Country of origin is listed next to each isolate according to their two-letter designation (CA, Canada; DE, Germany; ES, Spain; GB, Great Britain; IN, India; NL, Netherlands; US, United States). Green and blue bars indicate environmental and clinical isolates, respectively. Black and white bars represent mating type MAT1-1 and MAT1-2, respectively. Open red circles indicate azole resistant isolates. Solid red circles indicate pan-azole resistant isolates (i.e., isolates resistant to at least two different clinical azoles based on MIC testing). Red slash marks represent isolates without MIC test data for either itraconazole, voriconazole, or posaconazole. Red check marks indicate isolates with TR mutations. Yellow circles indicate the cytB G143A mutation conferring resistance to QoI fungicides. Purple circles indicate the benA F219Y mutation conferring resistance to MBC fungicides. Gray circles indicate the sdhB H270Y/R mutations conferring resistance to SDHI fungicides.

**Figure S2. ADMIXTURE analysis of K2-20.** Numbers within plots represent populations. Y-axis represents ancestry. A rectangular version of the neighbor-joining subsample tree (Figure 2) is on the x-axis. Black branches represent Clade 1. Red branches represent Clade 2. Blue branches represent Clade 3. Cross Validation values are shown in Figure S3.

**Figure S3. Cross validation.** Cross validation values for ADMIXTURE analysis shown in Figure S2.

**Figure S4. Gene tree of housekeeping genes and ITS region.** Gene trees with 729 isolates were constructed for *act1, efg1, gapdh1, histh4,* the ITS region*, rpb2, sdh1,* and *tef1.* Circle size on branches is a proxy for bootstrap values. Black squares represent isolates from Clade 1. Red squares represent isolates from Clade 2. Blue squares represent isolates from Clade 3.

**Table 1: Sampling of *A. fumigatus* strains from agricultural sites.**

**Table 2: Mutations associated with multi-fungicide resistance in azole-resistant *A. fumigatus.***

**Table S1. Metadata for US isolates.** Metadata for 431 US isolates used in map.

**Table S2: Metadata of all isolates.** Metadata for all 729 worldwide isolates used in this study.
